# iPSC‐derived human cardiac progenitor cells improve ventricular remodelling *via* angiogenesis and interstitial networking of infarcted myocardium

**DOI:** 10.1111/jcmm.12725

**Published:** 2015-11-27

**Authors:** KP Myu Mia Ja, Qingfeng Miao, Nicole Gui Zhen Tee, Sze Yun Lim, Manasi Nandihalli, Chrishan J.A. Ramachandra, Ashish Mehta, Winston Shim

**Affiliations:** ^1^ National Heart Research Institute SingaporeNational Heart Centre SingaporeSingapore; ^2^ Department of PharmacologyHebei Medical UniversityShijiazhuangChina; ^3^ DUKE‐NUS Graduate Medical School

**Keywords:** interstitial cells, cardiac progenitors, cardiomyocytes, telocytes, cardiac repair

## Abstract

We investigate the effects of myocardial transplantation of human induced pluripotent stem cell (iPSC)‐derived progenitors and cardiomyocytes into acutely infarcted myocardium in severe combined immune deficiency mice. A total of 2 × 10^5^ progenitors, cardiomyocytes or cell‐free saline were injected into peri‐infarcted anterior free wall. Sham‐operated animals received no injection. Myocardial function was assessed at 2‐week and 4‐week post‐infarction by using echocardiography and pressure‐volume catheterization. Early myocardial remodelling was observed at 2‐week with echocardiography derived stroke volume (SV) in saline (20.45 ± 7.36 μl, *P* < 0.05) and cardiomyocyte (19.52 ± 3.97 μl, *P* < 0.05) groups, but not in progenitor group (25.65 ± 3.61 μl), significantly deteriorated as compared to sham control group (28.41 ± 4.41 μl). Consistently, pressure**–**volume haemodynamic measurements showed worsening chamber dilation in saline (EDV: 23.24 ± 5.01 μl, *P* < 0.05; ESV: 17.08 ± 5.82 μl, *P* < 0.05) and cardiomyocyte (EDV: 26.45 ± 5.69 μl, *P* < 0.05; ESV: 18.03 ± 6.58 μl, *P* < 0.05) groups by 4‐week post‐infarction as compared to control (EDV: 15.26 ± 2.96 μl; ESV: 8.41 ± 2.94 μl). In contrast, cardiac progenitors (EDV: 20.09 ± 7.76 μl; ESV: 13.98 ± 6.74 μl) persistently protected chamber geometry against negative cardiac remodelling. Similarly, as compared to sham control (54.64 ± 11.37%), LV ejection fraction was preserved in progenitor group from 2‐(38.68 ± 7.34%) to 4‐week (39.56 ± 13.26%) while cardiomyocyte (36.52 ± 11.39%, *P* < 0.05) and saline (35.34 ± 11.86%, *P* < 0.05) groups deteriorated early at 2‐week. Improvements of myocardial function in the progenitor group corresponded to increased vascularization (16.12 ± 1.49/mm^2^ to 25.48 ± 2.08/mm^2^ myocardial tissue, *P* < 0.05) and coincided with augmented networking of cardiac telocytes in the interstitial space of infarcted zone.

## Introduction

Myocardial transplantation of stem cells remains a keenly investigated option for rejuvenating the failing heart in spite of the ambivalent outcome from clinical studies 1, 2. Recent reports of large‐scale transplantation of human embryonic stem cell (hESC)‐derived cardiac progenitors in heart failure (HF) patients 3 and cardiomyocytes in non‐human primates 4 have revived interest in cellular replacement therapy. However, it is currently far from clear what are the ideal cell types for cardiac regenerative therapy.

Bone marrow‐derived stem cells (BMSCs) and mesenchymal stem cells (MSCs) have been widely studied in many clinical and preclinical studies. Despite early promises, it is believed that transplanted BMSCs/MSCs do not differentiate into *bona fide* cardiomyocytes in a meaningful manner to replace heart muscle cells lost during myocardial infarction (MI), rather the beneficial outcome on cardiac performance have been generally attributed to paracrine effects 1, 5. Nevertheless, clinical results from myocardial biopsy derived‐cardiac stem cells (CSCs) 6 and cardiosphere‐derived cells (CDCs) 7 have been exciting, whereby the derived cardiovascular progenitors that are capable of differentiating into cardiomyocytes and endothelial cells are thought to contribute to new muscle formation and to induce robust neovascularization in the myocardium. In comparison to fully differentiated cardiomyocytes, cardiovascular progenitors are likely to be more resilient and to have greater adaptive responses to the hypoxic and anoxic milieu of the infarcred myocardium. This is consistent with the apparent robust recovery of left ventricular ejection fraction (LVEF) observed in the recipients of CSCs 6 than other clinical studies that utilized BMSCs/MSCs. Therefore, cardiovascular progenitors may be the ideal choice of cells for HF as compared to pluripotent stem cell‐derived cardiomyocytes that are clinically more challenging to prepare or BMSCs/MSCs that appeared less potent in improving contractile performance. Further investigations into cardiovascular progenitors are warranted as wider adoption of such cells may significantly leapfrog cardiac regenerative medicine.

There are limited studies that directly compare functional outcome of myocardial transplantation of cardiac progenitors and fully differentiated cardiomyocytes in repairing infarcted myocardium. In this study, we examined the effect of transplanting human induced pluripotent stem cell (hiPSC)‐derived cardiac progenitors and cardiomyocytes into acute infarcted myocardium in severe combined immune deficiency (SCID) mice and investigated their contribution to cardiac performance at 2‐week and 4‐week post‐transplantation. We report that both cardiac progenitor and cardiomyocyte transplantation exerted early protective effect on LV remodelling post‐infarction, cardiac progenitors additionally preserved cardiac contractile function by paracrine effects through enhanced angiogenesis and augmented networking with myocardial telocytes in the infarcted milieu.

## Materials and methods

### Generation of iPSC‐cardiac progenitors and cardiomyocytes

The iPSC cell line utilized was derived from neonatal human dermal fibroblasts for generation of cardiac progenitors and cardiomyocytes as reported previously 8. Briefly, the iPSCs were enzymatically passaged, plated on 1% matrigel‐coated dishes and maintained in mTeSR1 medium (Stemcell Technologies Inc, Vancouver, BC, Canada). Cardiac differentiation was initiated by embryoid body (EB) formation in the presence of 5 μM SB203580 for 8 days to reach cardiac progenitor stage for transplantation experiment. The day 8 EBs were additionally plated on gelatin‐coated dishes in EB medium without SB203580 to produce cardiomyocytes. Beating cluster of cardiomyocytes started to be observed by Day 12 and were dissected by Day 18 of differentiation for transplantation experiment.

### Flow cytometry

Flow cytometry analysis was performed as previously described 9. Briefly, day 8 EBs or Day 18 cardiomyocytes were made into single cells by dissociation using TrypLE (Thermo Fisher Scientific, Waltham, MA, USA). Cells were fixed and stained with PE‐conjugated Sirpa (BD Biosciences, Mississauga, ON, Canada), Nkx2.5 (Novus Biologicals, Littleton, CO, USA) and cardiac troponin T (cTNT; United States Biological, Salem, MA, USA) before incubating with appropriate Alexa Fluor 555 or 350‐conjugated secondary antibody (Thermo Fisher Scientific). Data were acquired and analysed on FACS Aria II (BD Biosciences). A total of 10,000 gated events were counted in three independent experiments.

### Real‐time PCR

RNA was isolated using RNeasy kit (Qiagen GmbH, Hilden, Germany) for real‐time reverse transcription PCR as previously described 9. Briefly, one microgram of total RNA was converted to cDNA and five nanogram of the cDNA template was amplified using Quantifast kit (Qiagen GmbH) using forward and reverse primers (Table S1). Relative quantification was computed by the ∆∆Ct method (using endogenous GAPDH as control) and day‐specific expression levels were normalized to its baseline values. Heat‐map was generated from normalized Ct values using Genesis software. All experiments were performed in triplicates.

### Animal surgery and transplantation

All animal procedures were approved by the Singapore General Hospital IACUC committee and conformed with the Guide for the Care and Use of Laboratory Animals published by the US National Institute of Health (NIH publication No.85‐23, revised 1996). The surgical procedures were described previously 10. Briefly, Healthy female mice with SCID (10–12 weeks of age, weighing 22–25 g) were used. A left thoracotomy was performed to permanently ligate the left anterior coronary artery using a 7‐0 suture (Ethicon; Johnson and Johnson, New Brunswick, NJ, USA). Immediately following coronary ligation, occlusion was confirmed by observation of LV pallor. Animals were randomly assigned to four groups: sham‐operated animals, saline‐injected animals that received PBS (50 μl) or cell‐injected animals that received 2 × 10^5^ cardiac progenitors or cardiomyocytes (50 μl) intramyocardially into the LV free wall bordering the infarct zone. Animals were weaned off the ventilator until fully recovered and given free access to standard chow and water until next follow‐up.

### Echocardiography and pressure**–volume catheterization**

Transthoracic echocardiography was performed at baseline, 2‐week and 4‐week after infarction on Vevo2100 (VisualSonics VSI, Toronto, ON, Canada) with MS400 linear array transducer (38 MHz) by using optimized sector width for complete myocardial visualization (50 and 110 μm axial and lateral resolution, respectively) and endocardial definition. A 2D guided M‐mode of parasternal short axis at papillary muscle level was obtained to measure standard parameters. An average of 10 cardiac cycles at each plane was stored in cineloop for subsequent offline analysis in a blinded manner. Ventricular chamber volumes and LVEF were derived by using a modified Quinones method 11 and LV fractional shortening was calculated as (LVIDed‐LVIDes) × 100%/LVIDed.

Pressure**–**volume haemodynamic parameters were gathered by a 1.4‐French microtip pressure**–**volume conductance catheter (Millar Instruments, Houston, TX, USA) through inserting into right carotid artery and advanced into the left ventricle into healthy control animals or before sacrifice of animals at 2‐week or 4‐week post‐infarction/treatment. The positioning of the catheter was visualized and guided by echocardiography. Calibration for parallel conductance volume and manual transient compression of inferior vena cava for derivation of load‐independent PV parameters was performed according to Pacher *et al*. 12. Data were analysed using PVAN3.2 (Millar Instruments).

### Histology and immunostaining

Harvested hearts were cryopreserved by using OCT Tissue‐Tek medium and sectioned transversely from the basal part to the apex of left ventricle using a cryostat with 5 μm thickness (Leica AG, Solms, Germany). Masson's Trichrome staining (Sigma‐Aldrich, St. Louise, MO, USA) was performed to quantify infarct size. The percentage infarct scar size was estimated from infarct area over total LV area using a calibrated M205 steromicroscope (Leica AG, Solms, Germany). Overnight incubation with antibody against human‐specific Ku80 (clone EPR3468; Abcam, Cambridge, MA, USA) was used to identify the transplanted human cardiac progenitors and cardiomyocytes. Antibodies against c‐kit (clone 104D2; Dako, Glostrup, Denmark) and sarcomeric α‐actinin (clone EA53; Sigma‐Aldrich) were used to identify myocardial telocytes and cardiac muscle cells, respectively. Signals visualization was performed with 3,3′‐diaminobenzidine (DAB) before counerstaining nuclei with haematoxylin. For vascular angiogenesis, microvessels of less than 200 μm caliber in the peri‐infarcted myocardium, away from the pericardium, were identified by using antibody against α‐smooth muscle actin (clone 1A4; Sigmal‐Aldrich) and Alexa Fluor488‐conjugated secondary antibody (Life Tecnologies, Carlsbad, CA, USA) before counterstaining the nuclei with 4′6‐diamidino‐2‐phenylindole and signals visualized and analysed on a micrometer calibrated M200 fluorescent microscope (Carl Zeiss, Gottingen, Germany). For co‐staining of Ku80 positive human cells in histology sections (visualized by using DAB colorimetric method), sequential staining with primary antibody against c‐kit or sarcomeric α‐actinin was followed by fluorescent secondary antibody.

### Statistical analysis

Data were presented as mean ± S.D. anova was performed comparing among experimental groups followed by post hoc analysis by Dunnett *t*‐test against control. For microvascular density counts, comparison between 2‐week and 4‐week was performed using Student's *t*‐test. Analysis was performed with SPSS software (version 13; SPSS Inc, Chicago, IL, USA) with *P* < 0.05 considered statistically significant.

## Results

### Characterization of hiPSC‐derived cardiac progenitors and cardiomyocytes

**Figure 1:**
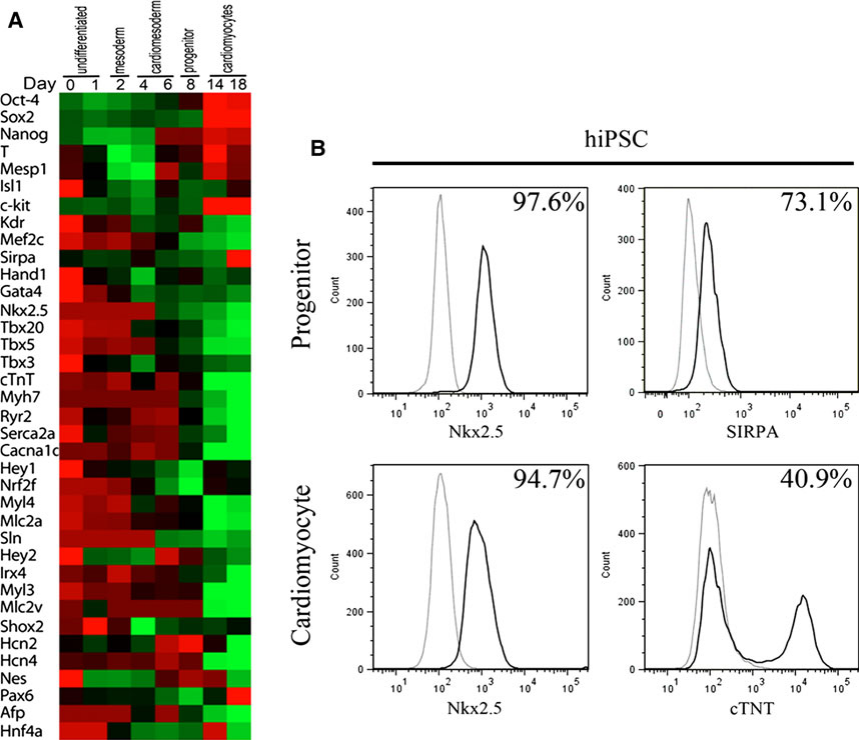
*In vitro* cardiac developmental ontogeny of human induced pluripotent stem cells. (**A**) Heat map representing temporal gene expression kinetics of MSnviPSNF3 hiPSC line. Normalized Ct values were plotted, where lower Ct values represent higher expression (green). Note the expression of progenitor markers at Day 8 (notably *c‐kit*,* Sirpa, Isl1 & Nkx2.5*) and cardiomyocyte markers post‐day 14 (notably *MLC2v, cTNT, HCN4, Cacna1c*) of differentiation. Data represent mean ±S.E.M. of three independent experiments. (**B**) Flow cytometry analysis of progenitors and cardiomyocytes showing high expression of Nkx2.5 and SIRPA in the progenitor stage and expression of Nkx2.5 and cTNT in the cardiomyocyte stage.

Pluripotency of the hiPSC line, MSnviPSNF3, used in this study was characterized previously 8. Gene expression profiling consistently showed that high levels expression of pluripotent markers of Oct‐4, Sox2 and Nanog were only apparent at undifferentiated and early mesoderm stages (Fig. 1A). Furthermore, following SB203580 induction and low serum embryoid bodies differentiation protocol 8, cardiac developmental ontogeny was recapitulated whereby gene expression signifying orderly transition from mesoderm (*e.g*. brachyury) to cardiomesoderm (*e.g*. Mesp1) to progenitor (*e.g*. Sirpa, Isl1) and finally to committed cardiomyocytes (*e.g*. troponin T, Myh7 and MLC2v) was observed (Fig. 1A). Moreover, expression of c‐kit, a key marker of myocardial telocytes 13, 14, 15 and CSCs 16, 17 was detected until cardiac progenitor stage at Day 8 of differentiation, but was not apparent by cardiomyocyte stage at Day 14 onwards.

Consistent with previous reports of membrane 18 and nuclear markers for cardiac progenitors 19, flow cytometry confirmed cell surface expression of SIRPA (73.1%) in our Day 8 cardiac progenitors that were expressing high levels of Nkx2.5 (97.6%) transcription factor (Fig. 1B). These progenitors differentiated into cardiomyocytes whereby approximately 95% of the cells maintained Nkx2.5 expression while about 41% expressed troponin T by Day 14 of differentiation.

### Integrin and laminin expression in cardiac progenitors and cardiomyocytes

**Figure 2:**
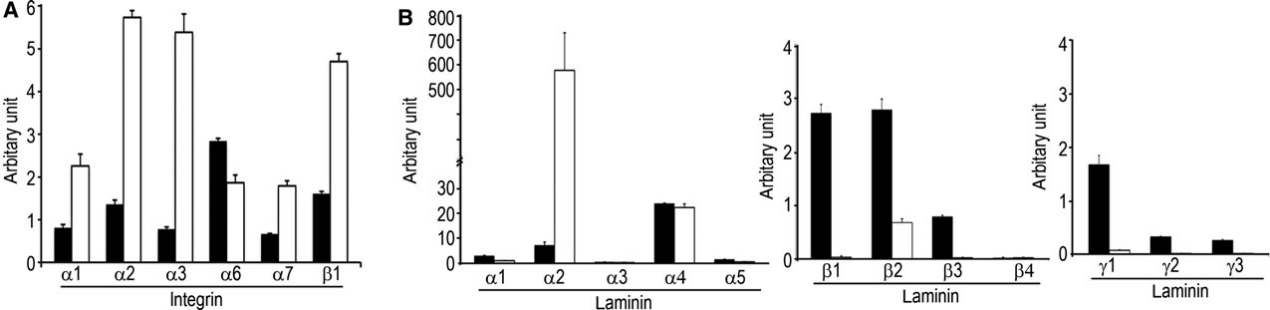
Characterization of integrin and laminin expression of progenitors and cardiomyocytes. (**A**) Increased cellular expression of major integrins for collagen and laminin matrices in the differentiated cardiomyocytes. (**B**) Distinct expression profile of laminin subunits during cardiac differentiation with laminin‐411/421 matrices pre‐dominant early in progenitors and a switch to pre‐dominantly laminin‐211/221 matrices later in cardiomyocytes. Data represent mean ± S.D. of three independent experiments.

A subset of integrins was found to specifically upregulated in cardiomyocytes as compared to cardiac progenitors. With the exception of α6 integrin, cardiomyocytes expressed substantially higher levels of α1, α2, α3, α7 and β1 integrins than cardiac progenitors (Fig. 2A). This is consistent with cardiac specification process whereby integrin family members for collagen (α1 & α2 integrins) 20 and laminin (α2, α3 & α7 integrins) 21, 22 becoming highly expressed in cardiomyocytes. Consistently, cardiac muscle laminins (laminin‐221 and laminin‐211) 22, 23 were majorly expressed in our Day 14 differentiated cardiomyocytes. In contrast, laminin‐411 and laminin‐421 that have been associated with vascularization 24 were more abundantly expressed in our Day 8 differentiated cardiac progenitors (Fig. 2B).

### Echocardiography assessment of effects of cell transplantation in acute myocardial infarction

Consistent with our previous results of transplantation of hMSCs into acutely infarcted rodent myocardium 10, intramyocardial cell injection benefited early cardiac remodelling by restricting ventricular dilation as demonstrated by 2‐week post‐infarction. Echocardiography of the infarcted myocardium revealed significant deterioration of ventricular dimension and chamber dilation in saline injected (LVIDed: 3.70 ± 0.47 mm, *P* < 0.05; ESV: 35.09 ± 20.65 μl, *P* < 0.05) group, but not the progenitor (LVIDed: 3.61 ± 0.32 mm; ESV: 29.26 ± 13.16 μl) or cardiomyocyte (LVIDed: 3.61 ± 0.16 mm; ESV: 33.68 ± 2.04 μl) injected groups when compared to control (LVIDed: 3.19 ± 0.29 mm; ESV: 15.27 ± 4.90 μl) group (Tables S1 and S2). Nevertheless, the protective effects of cell transplantation failed to sustain by 4‐week of infarction whereby LV chamber dilation in progenitor (LVIDed: 4.21 ± 0.58 mm, *P* < 0.05; ESV: 53.17 ± 11.07 μl, *P* < 0.05) or cardiomyocyte (LVIDed: 4.25 ± 0.14 mm, *P* < 0.05; 53.17 ± 11.07 μl, *P* < 0.05) deteriorated similarly to saline injected (LVIDed: 3.93 ± 0.58 mm, *P* < 0.05; ESV: 47.22 ± 27.99 μl, *P* < 0.05) group when compared to control (Tables S1 and S2).

Similarly, beneficial effects of cell transplantation on stroke volume (SV) and LVEF was demonstrated at 2‐week initially, but subsided by 4‐week post‐infarction. However, functional protection was observed only in progenitor (SV: 25.65 ± 3.61 μl; LVEF: 48.94 ± 14.96%), but not the cardiomyocyte‐(SV: 19.52 ± 3.97 μl, *P* < 0.05; EF: 36.53 ± 5.84%, *P* < 0.05) or saline‐(SV: 20.45 ± 7.36 μl, *P* < 0.05; LVEF: 32.97 ± 18.35%, *P* < 0.05) injected groups when compared to control (SV: 28.41 ± 4.41 μl; LVEF: 65.43 ± 8.80%) group (Table S1).

### Pressure**–volume haemodynamic assessment of cell transplantation post‐infarction**

Consistent with chamber dilation at diastole by echocardiography early post‐infarction, deteriorated LV diastolic relaxation was observed only in the saline (EDPVR: 1.00 ± 0.45 mmHg. μl, *P* < 0.05) injected group as compared to control (EDPVR: 0.47 ± 0.19 mmHg/μl) group at 2‐week post‐infarction (Table S3). Furthermore, chamber volume at diastole and at systole worsened in both the saline‐(EDV: 23.24 ± 5.01 μl, *P* < 0.05; ESV: 17.08 ± 5.82 μl, *P* < 0.05) and cardiomyocyte‐(EDV: 26.45 ±5.69 μl, *P* < 0.05; ESV: 18.03 ± 6.58 μl, *P* < 0.05) injected groups by 4‐week post‐infarction as compared to control (EDV: 15.26 ± 2.96 μl; ESV: 8.41 ± 2.94 μl) group (Table S4). In contrast, cardiac progenitors protected against negative cardiac remodelling whereby chamber volume was preserved until 4‐week (EDV: 20.09 ± 7.76 μl; ESV: 13.98 ± 6.74 μl) post‐infarction. Furthermore, LVEF was protected early at 2‐week post‐infarction in cardiac progenitor group (LVEF: 38.68 ± 7.34%), but not the cardiomyocyte‐(LVEF: 36.52 ± 11.39%, *P* < 0.05) and saline‐(LVEF: 35.34 ± 11.86%, *P* < 0.05) injected groups and remained depressed in the saline (LVEF: 34.13 ± 10.88%, *P* < 0.05) group at 4‐week post‐infarction when compared to control (LVEF: 54.64 ± 11.37%) group (Tables S3 and S4).

### Infarct size after cell transplantation

**Figure 3:**
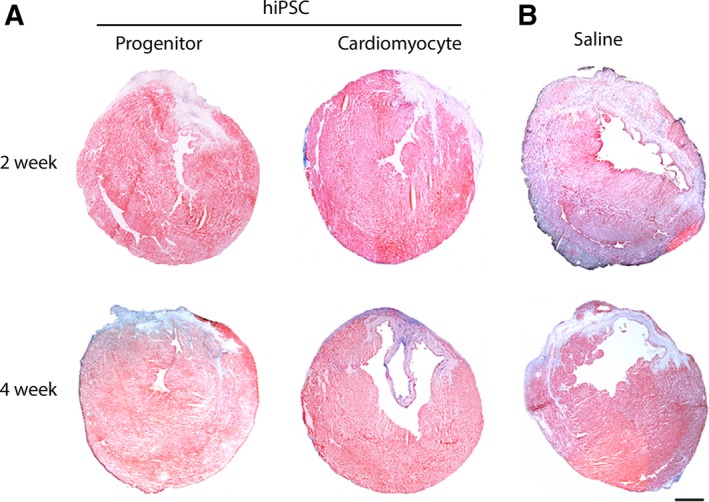
Infarct size estimation with Masson's trichrome staining. (**A**) Infarcted mouse heart received progenitor and cardiomyocyte injection showed thinning of anterior myocardial wall and transmural fibrotic remodelling. (**B**) Saline‐injected mouse heart showing thinned ventricular wall and extension of fibrosis into adjacent peri‐infarct regions; scale bar: 1 mm.

Consistent with our previous results of injecting human MSCs into infarcted mouse heart 10, cell transplantation in the current study similarly failed to reduce infarct size at 4‐week post‐infarction. The size of transmural infarct in progenitor (2‐week: 17.4 + 4.0%; 4‐week: 17.8% + 4.1%) and cardiomyocyte (2‐week: 23.9 + 10.3%; 4‐week 14.0 + 3.3%) transplanted groups (Fig. 3A) was not statistically different than the saline‐injected (2‐week: 22.4 + 8.5%, *P* = ns; 4‐week: 24.1% + 12.4%, *P* = ns) group (Fig. 3B) at the subacute phase of 2‐week or by 4‐week post‐cell injection.

### Integration of transplanted progenitors and cardiomyocytes

**Figure 4:**
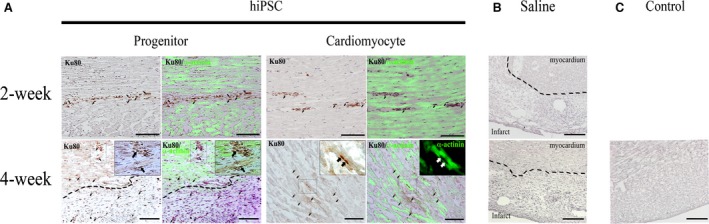
Localization of transplanted human iPSC‐derived progenitors and cardiomyocytes in myocardium. (**A**) Human‐specific nuclear staining of Ku80 in transplanted progenitors (arrows) and cardiomyocytes (arrows) in the interstitial space of α‐actinin (green) stained cardiac muscle at 2‐week post‐injection in the peri‐infarcted myocardium. Progenitor: Peri‐vascular (boxed) and interstitial (demarcated line) localization of Ku80 positive progenitors (arrows) in the infarct and peri‐infarct zone at 4‐week post‐injection. Magnified view of boxed region, showing Ku80 stained progenitors (arrows) located in proximity of vascular structures. Cardiomyocyte: Engraftment of Ku80 positive human cardiomyocytes (arrows) into the cardiac muscle fibers (boxed) of the peri‐infarcted myocardium. Magnified view of boxed region, showing Ku80 stained nuclei of human cardiomyocytes (arrows) revealing sarcomeric cardiac cross‐striations of α‐actinin. (**B**) Saline‐injected groups show no Ku80 staining in the myocardium of peri‐infarct or infarct region (demarcated line) at 2‐week or 4‐week post infarction. (**C**) Control group shows no infarction and no Ku80 staining. Dotted lines demarcates intact myocardium form infarcted zone; scale bar: 100 μm.

In contrast with saline injection and control groups, immunohistochemical staining with human‐specific Ku80 antibody revealed that both transplanted progenitors and cardiomyocytes resided along needle‐injection track at 2‐week post injection into the peri‐infarcted zone (Fig. 4A top panel arrows). However, the Ku80 stained human cardiac progenitors were majorly found in the infarcted zone that were devoid of α‐actinin stained cardiac muscle bundles at 4‐week post‐infarction with no appreciable cardiac differentiation detected (Fig. 4A bottom panel arrows). Nevertheless, some transplanted progenitors were located in proximity to microvascular structures in viable muscle layer (Fig. 4A bottom panel inset) and in the infarcted zone (Fig. 4A bottom panel arrows) by 4‐week post infarction. In contrast, the injected human cardiomyocytes remained in the peri‐infarcted zone and resided in close proximity to viable muscle fibres at 4‐week post‐infarction (Fig. 4A bottom panel arrows). Furthermore, a limited number of the transplanted Ku80 positive human cardiomyocytes were found to engraft into α‐actinin stained cross‐striated cardiac muscle fibres (Fig. 4A bottom panel inset).

**Figure 5:**
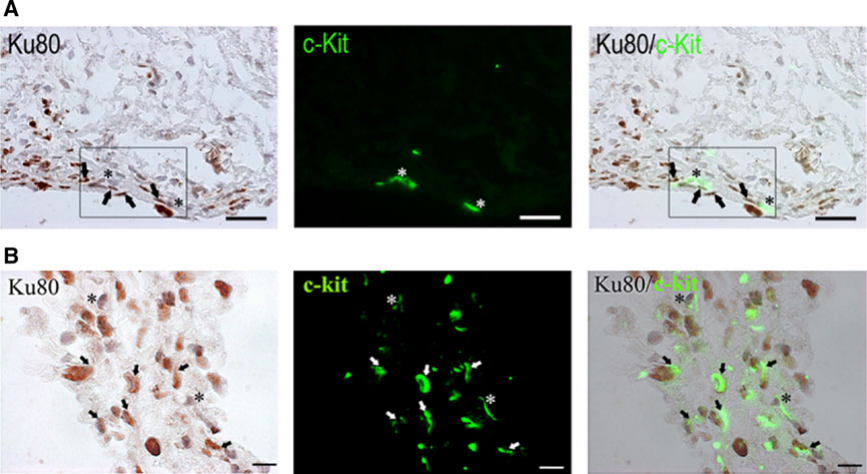
Presence of telocytes in the infarct region of the myocardium. (**A**) Human‐specific nuclear staining of Ku80 shows transplanted human progenitors (arrows) in close physical proximity (boxed) with c‐kit (green) stained (but human Ku80 negative) resident cardiac telocytes (asterisks). (**B**) Dual positive staining of human‐specific Ku80 and c‐kit in the transplanted human progenitors (arrows) that intermixed with c‐kit (green) positive (but Ku80 negative) resident cardiac telocytes (asterisks) in the infarcted region of the myocardium; scale bar: 50 μm (for **A**), 20 μm (for **B**).

Consistent with our previous reported presence of telocytes in the interstitial space of infarcted zone of myocardium 10, some c‐kit positive, but human Ku80 negative, telocytes (Fig. 5A asterisks) with long cellular processes were found in the interstitial space in proximity to transplanted human progenitors (Fig. 5A arrows) at 4‐week post‐infarction. Nevertheless, c‐kit and human Ku80 dual positive human cells were also found in the infarcted zone (Fig. 5B arrows). In contrast, such presence of c‐kit positive cells was not detected in the cardiomyocyte transplanted group.

### Progenitor transplantation enhances neovascularization

**Figure 6:**
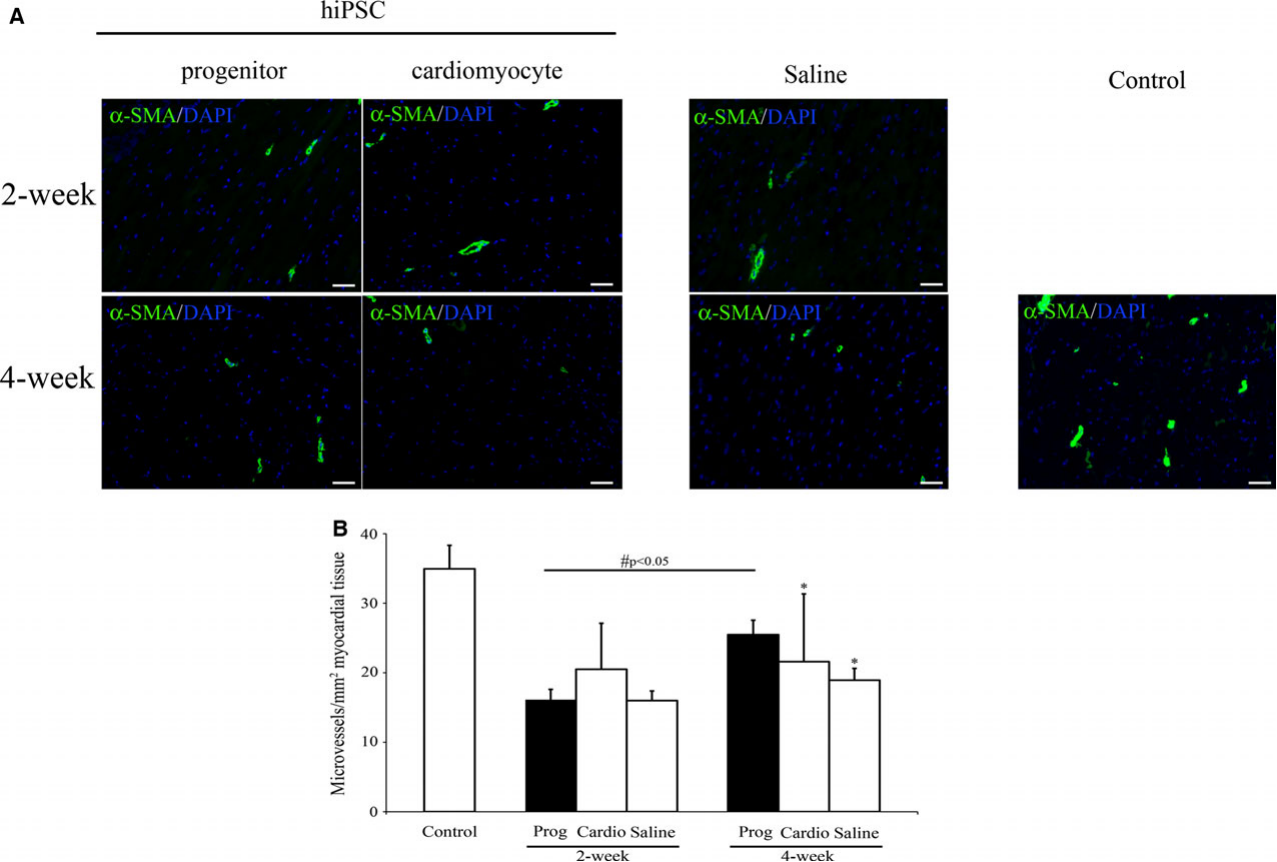
Microvascular neoangiogenesis at 2‐week and 4‐week post‐myocardial infarction. Immunofluorescent staining for microvasculature positive for α‐smooth muscle actin (SMA) in left ventricle post‐infarction. (**B**) Microvascular density counts of α‐SMA stained microvessels in the left ventricle. *P < 0.05 *versus* control; #*P* < 0.05 progenitor (2‐week *versus* 4‐week); scale bar: 20 μm.

During sub‐acute phase at 2‐week post‐infarction, reduced microvascularity in both the cardiac progenitor and cardiomyocyte transplanted groups was observed. The depressed microvascularity was demonstrated by reduced α‐smooth muscle actin stained microvasculature (Fig. 6A). Furthermore, there were no significant differences in microvascular density counts among the progenitor, cardiomyocyte or saline injection groups at 2‐week post‐infarction (Fig. 6B). However, microvasculature recovered in the progenitor group, but not in cardiomyocyte‐or saline‐injected group, whereby microvascular density counts were significantly enhanced from 16.12 ± 1.49/mm^2^ to 25.48 ± 2.08/mm^2^ myocardial tissue by 4‐week in animals that received progenitor transplantation. In contrast, microvascular density counts in cardiomyocyte (21.59 ± 9.75/mm^2^, *P* < 0.05) and saline (18.92 ± 1.70/mm^2^, *P* < 0.05) groups remained significantly depressed in comparison to the control (34.95 ± 3.38/mm^2^) group at 4‐week post‐infarction (Fig. 6B).

## Discussion

Ischaemic heart disease and its sequelae of MI remain a leading cause of cardiac mortality and morbidity. Despite optimal medical therapy and aggressive revascularization efforts in current cardiology practice, approximately 20% of MI survivors spiral towards congestive HF and face a grim fact of 5‐year mortality rate of 50% 25.

Over the past two decades, cellular‐based therapy has emerged as a potential new therapy for patients with advanced HF. Meta‐analysis of past clinical trials, majority of which involved BMSCs, have supported a potential benefit in treating HF 26, 27. Major commercial entities continue sponsoring late phase trials such as Capricor Therapeutics (phase II for CDCs), Athersys (phase II for MultiStem), Caladrius Biosciences (phase II for NBS10), Vericel (phase IIb for ixmyelocel‐T), Celyad (phase III for C‐Cure) and Mesoblast (phase III for mesenchymal precursor cells) 25. This indicates that cell therapy for myocardial repair remains very much a focus of the cardiovascular communities. The utilized cells included BMSCs that were primed for cardiac differentiation or cardiac biopsy derived cardiac resident stem cells (CDCs/CSCs). Nevertheless, it remains far from clear what are the ideal cell types for cardiac regenerative therapy for HF. Indeed, the first myocardial grafting of hESC‐derived cardiomyocytes was reported in HF patient recently, indicating that additional understanding of basic cellular biology involved in cell therapy is still warranted.

In this study, we investigated the effect of transplanting hiPSC‐derived cardiac progenitors and cardiomyocytes into MI rodent model. Transplantation of cardiac progenitors in the acute infarcted myocardium preserved chamber geometry and restricted ventricular remodelling. In contrast, transplantation of cardiomyocytes failed to significantly improve cardiac remodelling. Similarly, intramyocardial injection of hESC‐derived cardiomyocytes into chronically infarcted rat myocardium failed to arrest ongoing remodelling despite successful engraftment 28. Such findings on the beneficial effect of cardiac progenitors were in agreement with pre‐clinical studies using cardiac progenitor cell populations reported in cardiomyocyte progenitor cells (CMPCs) 29, CSCs 16 and CDCs 17. In fact, subsequent clinical studies using CSCs (SCIPIO) 6 and CDCs (CADUCEUS) 7 were reported to increase cardiac function including greater than 10% global LVEF improvement in selected patients in the SCIPIO trial at 12‐month 30.

Myocardial transplanted progenitors localized favourably to vascular structures (Fig. 4A bottom panel inset) and interacted intimately with c‐kit positive telocytes in the infarct and peri‐infarct regions. Consistently, we previously reported presence of telocytes in the infarct region of human MSC transplanted heart in a mouse infarction model 10. However, telocytes that were nursing closely to the transplanted progenitors were observed only in 4‐week, but not 2‐week post‐MI that was consistent with previous findings of diminishing presence of telocytes early post‐MI 31. Although their present did not ameliorate infarct size in current study, such recovery window of myocardial telocytes coincided with increased angiogenesis observed in the progenitor transplanted group from 2‐week to 4‐week post‐MI.

In contrast with cardiomyocytes that express muscle‐specific laminin‐211/221 32, our cardiac progenitors were found to express laminin‐411/421 *in vitro*. These laminins are known to support endothelial vascularization 23. Telocytes are known to interact with CSCs and to contribute to robust angiogenesis in infarcted myocardium 33, however, it is unclear if expression of laminin‐411/421 by the progenitors had any added role in the observed interaction with telocytes and in contributing to enhanced angiogenesis and their peri‐vascular localization. Moreover, the presence of cells with long processes that stained dual positive for Ku80 and c‐kit suggested that some telocytes are likely of human origin, though no such telocytes in the myocardium of cardiomyocyte transplanted group were detected. Furthermore, presence of exogenous and endogenous telocytes was consistent with our previous observation in infarcted mouse heart transplanted with hiPSC‐derived MSCs 10. Therefore, human telocytes arising from c‐kit positive cardiac progenitor populations *in vitro* cannot be discounted, though its expression was undetected by Day 14. Nevertheless, our results suggested that telocytes may have played a unique role in contributing to functional recovery observed in the progenitor transplanted group. Indeed, telocytes are known to mediate inter‐cellular signalling through secreting cytokine/chemokine factors 34 or releasing extracellular vesicles 35 that may contain proangiogenic microRNAs post MI 36 besides acting as mechano‐sensing cells 37 or anatomical supporting cells in the myocardium 10, 38.

There were no major differences in cellular localization of the transplanted human cells observed at 2‐week between progenitor and cardiomyocyte transplanted groups. However, progenitors were pre‐dominantly distributed in the interstitial space of infarcted epicardial region while only laminin‐221/211‐expressing cardiomyocytes retained and engrafted around myofibres in the peri‐infarct region by 4‐week post MI. Consistently, transplanted hESC‐CMs were previously reported to localize to laminin‐211 matrices of infarcted myocardium 32. Such muscle localization pattern coincided with the higher expression of α2β1, α3β1 and α7β1 integrins in our cardiomyocytes *in vitro* where α3 and α7 integrins 22 are known to be the major binding partners for laminin that are abundant in the infarcted myocardium 32. In particular, α7 integrin has been known to bind with laminin‐2 and‐4 in muscle 39.

Cardiomyocyte transplantation protected ventricular remodelling transiently at 2‐week, even though only limited quantities of cardiomyocytes were detected by 4‐week post MI in our mouse model. This was consistent with experience observed in hESC‐CMs that showed beneficial effect in spite of low cellular retention 40 thereby reinforces that paracrine effects were likely involved in ventricular remodelling early after MI. Similarly, the lack of notable differentiation of transplanted progenitors towards cardiomyocytes in the infarcted myocardium despite improved cardiac performance observed also supported paracrine contribution of both progenitor and cardiomyocyte transplantation. However, progenitor transplantation persistently protected against chamber dilation indicating additional beneficial contribution from increased angiogenesis and presence of telocytes. Consistently, short‐term transplantation of CMPCs similarly revealed beneficial paracrine effect by maintaining vascular homeostasis and attenuating ventricular remodelling post MI 29. This couples with major logistic efforts in producing substantial numbers of hESC‐cardiomyocytes (about one billion cells/recipient) for transplanting into heart of non‐human primate that evoked cardiac arrhythmia 4, 41, lends support to adequacy of transplanting cardiac progenitors in protecting damaged myocardium post‐acute MI.

The cost‐benefit of progenitor transplantation was demonstrated in early and persistent protection against remodelling and dilating chamber as demonstrated by better preserved LV chamber dimensions and volume. Furthermore, progenitor transplantation better preserved SV and LVEF at 2‐week post‐MI. In contrast, SV and LVEF in the cardiomyocyte and saline groups were persistently depressed from 2‐week and continued until 4‐week post‐MI as assessed by echocardiography. However, it is unclear if proliferation of resident CSCs 42 or moderation of tissue stiffness 43 and telocyte mediated restoration of tissue elasticity 14, 44 were also contributing to the observed beneficial effects by cardiac progenitor transplantation.

Collectively, our results show that localized injection of hiPSC‐derived cardiac progenitor cells alleviates ventricular remodelling and protects myocardial function by improving angiogenesis and enhancing interstitial cellular networking in the infarcted myocardium.

## Funding

This study was supported by funding from the National Research Foundation Singapore (NRF‐CRP‐2008‐02), the Goh Foundation (Duke‐NUS‐GCR/2013/008, GCR/2013/0010, GCR/2013/0011), the Biomedical Research Council Singapore (BMRC13/1/96/19/686A) and the SingHealth Foundation (SHF/FG569P/2014, SHF/FG630S/2014). The authors wish to thank Glen Lester Sequiera and Yuliansa Sudibyo for assisting in routine maintenance of hiPSC culture, Dr. Shiqi Li and Ting Huay Ooi for assisting in animal experimentations, Cai Hong Koh and Pearly AJ Yong for help in flow cytometry analysis.

## Conflicts of interest

The authors confirm that there are no conflicts of interest.

## Supporting information


**Table S1** Echocardiography analysis of cardiac performance at 2‐week post‐intervention.
**Table S2** Echocardiography analysis of cardiac performance at 4‐week post‐intervention.
**Table S3** Haemodynamic performance by cardiac catheterization at 2‐week post‐intervention.
**Table S4** Haemodynamic performance by cardiac catheterization at 4‐week post‐intervention.
**Table S5** List of primers.
