# Supplementary material for: iPSC‐derived human cardiac progenitor cells improve ventricular remodelling via angiogenesis and interstitial networking of infarcted myocardium
Source: J Cell Mol Med. 2015 Nov 27;20(2):323–32. doi: 10.1111/jcmm.12725 (PMC4727567; doi:10.1111/jcmm.12725)
Supplement: Supplementary file 1 — Table S1 Echocardiography analysis of cardiac performance at 2‐week post‐intervention. Table S2 Echocardiography analysis of cardiac performance at 4‐week post‐intervention. Table S3 Haemodynamic performance by cardiac catheterization at 2‐week post‐intervention. Table S4 Haemodynamic performance by cardiac catheterization at 4‐week post‐intervention. Table S5 List of primers. [file JCMM-20-323-s001.docx]

| **Echocardiography** | **2-week follow-up** | | | | **p** |
| --- | --- | --- | --- | --- | --- |
|  | Control  (n=8) | Progenitor  (n=7) | Cardiomyocyte  (n=4) | Saline  (n=8) |  |
| Anted (mm) | 0.87 ± 0.08 | 0.95 ± 0.11 | 1.01 ± 0.06 | 0.89 ± 0.09 | NS |
| Antes (mm) | 1.38 ± 0.10 | 1.34 ± 0.13 | 1.31 ± 0.08 | 1.26 ± 0.21 | NS |
| LVIDed (mm) | 3.19 ± 0.29 | 3.61 ± 0.32 | 3.61 ± 0.16 | 3.70 ± 0.47# | #p<0.05 vs. Control |
| LVIDes (mm) | 1.66 ± 0.37 | 2.48 ± 0.48# | 2.54 ± 0.41# | 2.63 ± 0.49# | #p<0.05 vs. Control |
| LV chamber area Ed | 18.91 ± 1.56 | 21.74 ± 2.76 | 21.16 ± 0.53 | 23.35 ± 4.84# | #p<0.05 vs. Control |
| LV chamber area Es | 10.16 ± 1.93 | 14.74 ± 4.05 | 15.74 ± 0.58 | 15.45 ± 5.41 | NS |
| EDV (μL) | 43.71 ± 6.36 | 54.96 ± 10.52 | 53.20 ± 2.66 | 55.55 ± 15.03 | NS |
| ESV (μL) | 15.27 ± 4.90 | 29.26 ± 13.16 | 33.68 ± 2.04 | 35.09 ± 20.65# | #p<0.05 vs. Control |
| sLVEF (%) | 65.43 ± 8.80 | 48.94 ± 14.96 | 36.53 ± 5.84# | 32.97 ± 18.35# | #p<0.05 vs. Control |
| FS (%) | 48.51 ± 7.74 | 31.62 ± 8.64# | 29.90 ± 8.67# | 29.20 ± 6.47# | #p<0.05 vs. Control |
| SV (μL) | 28.41 ± 4.41 | 25.65 ± 3.61 | 19.52 ± 3.97# | 20.45 ± 7.36# | #p<0.05 vs. Control |

Supplemental Table 1. Echocardiography analysis of cardiac performance at 2-week post intervention.

| **Echocardiography** | **4-week follow-up** | | | | **p** |
| --- | --- | --- | --- | --- | --- |
|  | Control  (n=8) | Progenitor  (n=7) | Cardiomyocyte  (n=4) | Saline  (n=8) |  |
| Anted (mm) | 0.87 ± 0.08 | 0.87 ± 0.20 | 0.70 ± 0.14 | 0.88 ± 0.23 | NS |
| Antes (mm) | 1.38 ± 0.10 | 1.26 ± 0.30 | 0.86 ± 0.28# | 1.24 ± 0.37 | #p<0.05 vs. Control |
| LVIDed (mm) | 3.19 ± 0.29 | 4.21 ± 0.58# | 4.25 ± 0.14# | 3.93 ± 0.58# | #p<0.05 vs. Control |
| LVIDes (mm) | 1.66 ± 0.37 | 3.22 ± 0.87# | 3.58 ± 0.28# | 3.02 ± 0.85# | #p<0.05 vs. Control |
| LV chamber area Ed | 18.91 ± 1.56 | 24.35 ± 5.96 | 25.18 ± 1.60 | 23.35 ± 4.84 | NS |
| LV chamber area Es | 10.16 ± 1.93 | 19.57 ± 7.23 | 20.74 ± 2.79 | 18.41 ± 6.48 | NS |
| EDV (μL) | 43.71 ± 6.36 | 69.12 ± 28.29 | 71.06 ± 5.75 | 65.52 ± 23.31 | NS |
| ESV (μL) | 15.27 ± 4.90 | 50.08 ± 31.51# | 53.17 ± 11.07# | 47.22 ± 27.99# | #p<0.05 vs. Control |
| sLVEF (%) | 65.43 ± 8.80 | 32.66 ± 18.05# | 25.69 ± 11.35# | 32.97 ± 18.35# | #p<0.05 vs. Control |
| FS (%) | 48.51 ± 7.74 | 24.94 ± 10.80# | 15.77 ± 4.56# | 24.08 ± 12.05# | #p<0.05 vs. Control |
| SV (μL) | 28.41 ± 4.41 | 19.05 ± 5.60# | 17.89 ± 6.46# | 18.30 ± 6.46# | #p<0.05 vs. Control |

Supplemental Table 2. Echocardiography analysis of cardiac performance at 4-week post intervention.

| **Pressure-Volume loops** | **2-Week follow-up** | | | | **p** |
| --- | --- | --- | --- | --- | --- |
|  | Control  (n=7) | Progenitor  (n=3) | Cardiomyocyte  (n=5) | Saline  (n=6) |  |
| ESPVR (mmHg/μL) | 10.15 ± 2.42 | 11.082 ± 8.36 | 8.16 ± 2.22 | 7.04 ± 4.87 | NS |
| EDPVR (mmHg/μL) | 0.47 ± 0.19 | 0.53 ± 0.22 | 0.83 ± 0.44 | 1.00 ± 0.45# | #p<0.05 vs. control |
| PRSW (mmHg) | 50.01 ± 15.30 | 31.28 ± 11.47 | 31.84 ± 7.34 | 32.18 ± 13.63 | NS |
| dP/dtmax.EDV (mmHg/s/μL) | 376.34 ± 182.67 | 399.39 ± 335.88 | 274.12 ± 30.78 | 323.86 ± 172.53 | NS |
| SW (mmHg x μL) | 618.81 ± 203.22 | 570.37 ± 29.97 | 437.42 ± 154.92 | 471.20 ± 169.26 | NS |
| CO (uL/min) | 3158.14 ± 799.14 | 3579.00 ± 553.14 | 3134.20 ± 1324.84 | 2944.00 ± 806.44 | NS |
| SV (μL) | 8.20 ± 1.83 | 8.28 ± 0.28 | 7.58 ± 3.14 | 7.20 ± 1.60 | NS |
| ESV (μL) | 8.41 ± 2.94 | 15.35 ± 4.82 | 14.94 ± 6.02 | 16.36 ± 7.51 | NS |
| EDV (μL) | 15.26 ± 2.96 | 22.77 ± 4.16 | 21.26 ± 8.89 | 22.34 ± 7.01 | NS |
| ESP (mmHg) | 98.69 ± 19.97 | 98.89 ± 16.79 | 93.99 ± 5.79 | 100.71 ± 15.06 | NS |
| EDP (mmHg) | 17.53 ± 7.93 | 7.06 ± 2.58 | 20.02 ± 6.56 | 24.56 ± 9.54 | NS |
| EF (%) | 54.64 ± 11.37 | 38.68 ± 7.34 | 36.52 ± 11.39# | 35.34 ± 11.86# | #p<0.05 vs. Control |
| dP/dtmax (mmHg/s) | 6328.28 ± 1887.46 | 8022.67 ± 2192.92 | 5252.20 ± 1174.20 | 5886.50 ± 1783.63 | NS |
| dP/dtmin (mmHg/s) | -5565.14 ± 1773.84 | -6846.00 ± 1886.41 | -3741.40 ± 901.98 | -4957.33 ± 1074.39 | NS |
| dV/dtmax (μL/s) | 484.30 ± 168.98 | 512.93 ± 171.52 | 406.00 ± 224.39 | 569.35 ± 220.47 | NS |
| dV/dtmin (μL/s) | -234.54 ± 59.07 | -272.57 ± 24.76 | -332.78 ± 161.55 | -287.53 ± 55.36 | NS |
| Tau-Weiss (ms) | 10.69 ± 2.26 | 8.23 ± 1.11 | 15.17 ± 1.81# | 12.35 ± 2.50 | #p<0.05 vs. Control |

Supplemental Table 3. Hemodynamic performance by cardiac catheterization at 2-week post intervention.

| **Pressure-volume loops** | **4-week follow-up** | | | | **p** |
| --- | --- | --- | --- | --- | --- |
|  | Control  (n=7) | Progenitor  (n=6) | Cardiomyocyte (n=4) | Saline  (n=7) |  |
| ESPVR (mmHg/μL) | 10.15 ± 2.42 | 8.82 ± 3.10 | 10.11 ± 7.18 | 7.40 ± 2.12 | NS |
| EDPVR (mmHg/μL) | 0.47 ± 0.19 | 0.79 ± 0.41 | 0.84 ± 0.27 | 0.76 ± 0.44 | NS |
| PRSW (mmHg) | 50.01 ± 15.30 | 34.42 ± 13.74 | 38.73 ± 10.04 | 37.20 ± 17.60 | NS |
| dP/dtmax.EDV (mmHg/s/μL) | 376.34 ± 182.67 | 216.95 ± 126.70 | 333.84 ± 172.71 | 262.49 ± 170.31 | NS |
| SW (mmHg x μL) | 618.81 ± 203.22 | 404.42 ± 248.28 | 452.250 ± 97.954 | 400.07 ± 122.16 | NS |
| CO (uL/min) | 3158.14 ± 799.14 | 2956.43 ± 1229.02 | 3518.00 ± 663.68 | 2783.00 ± 862.37 | NS |
| SV (μL) | 8.20 ± 1.83 | 7.64 ± 3.14 | 9.99 ± 2.02 | 7.49 ± 1.99 | NS |
| ESV (μL) | 8.41 ± 2.94 | 13.98 ± 6.74 | 18.03 ± 6.58# | 17.08 ± 5.82# | #p<0.05 vs. Control |
| EDV (μL) | 15.26 ± 2.96 | 20.09 ± 7.76 | 26.45 ± 5.69# | 23.24 ± 5.01# | #p<0.05 vs. Control |
| ESP (mmHg) | 98.69 ± 19.97 | 88.67 ± 19.95 | 76.75 ± 8.78 | 82.41 ± 8.23 | NS |
| EDP (mmHg) | 17.53 ± 7.93 | 18.06 ± 4.48 | 21.65 ± 6.56 | 18.50 ± 9.53 | NS |
| EF (%) | 54.64 ± 11.37 | 39.56 ± 13.26 | 40.68 ± 10.87 | 34.13 ± 10.88# | #p<0.05 vs. Control |
| dP/dtmax (mmHg/s) | 6328.28 ± 1887.46 | 4391.86 ± 2155.42 | 4157.25 ± 1707.28 | 4464.57 ± 1495.53 | NS |
| dP/dtmin (mmHg/s) | -5565.14 ± 1773.84 | -3772.86 ± 2240.21 | -3861.00 ± 1582.14 | -3777.00 ± 1085.60 | NS |
| dV/dtmax (μL/s) | 484.30 ± 168.98 | 489.33 ± 275.92 | 742.40 ± 432.09 | 581.90 ± 246.31 | NS |
| dV/dtmin (μL/s) | -234.54 ± 59.07 | -497.98 ± 375.72 | -356.80 ± 128.88 | -258.50 ± 77.87 | NS |
| Tau-Weiss (ms) | 10.69 ± 2.26 | 21.14 ± 11.47# | 13.98 ± 4.91 | 12.69 ± 2.61 | #p<0.05 vs. Control |

Supplemental Table 4. Hemodynamic performance by cardiac catheterization at 4-week post intervention.

**Supplementary Table 5:** List of primers

| **Gene name** | **Forward primer** | **Reverse primer** |
| --- | --- | --- |
| *Pou5f1* | AGTTTGTGCCAGGGTTTTTG | ACTTCACCTTCCCTCCAACC |
| *Sox2* | AAAAATCCCATCACCCACAG | GCGGTTTTTGCGTGAGTGT |
| *Nanog* | CTCCATGAACATGCAACCTG | GAGGAAGGATTCAGCCAGTG |
| *T* | GCGCGAGAACAGCACTACTA | GACCAAGACTGTCCCCGCTC |
| *Mesp1* | CGAGTCCTGGATGCTCTCTG | CCATGAGTCTGGGGACGAGA |
| *Isl1* | AAGGACAAGAAGAGAAGCAT | CATGGGAGTTCCTGTCATCC |
| *c-Kit* | CAAGCACAATGGCACGGTTG | GGGGATGGATTTGCTCTTTGTT |
| *Kdr* | GGCGGCACGAAATATCCTCT | GGAGGCGAGCATCTCCTTTT |
| *Mef2c* | TGGAGAAGCACTTCAACGCT | TCCTGCATTCGTTCCTGATGA |
| *Sirpa* | TGCCAGAGAAATAACACAGGACA | CTGGCATACTCCGTGTGGTT |
| *Hand1* | GGAGTCCGCAGAAGGGTTAAA | CGGGCAAGGCTGAAAATGAG |
| *Gata4* | CGACACCCCAATCTCGATATGTT | ACAGATAGTGACCCGTCCCA |
| *Nkx2.5* | CAAGTGTGCGTCTGCCTTTC | CGCGCACAGCTCTTTCTTTT |
| *Tbx20* | AACCCCAAATCGAGGGTCAG | AAGAGCAGTCAGGGACTGTG |
| *Tbx5* | AGACCTGGCCTAAAGAGGTC | CGCAAGGTTCTGCTCTCCAA |
| *Tbx3* | TTTGAAGACCATGGAGCCCG | ACATTCGCCTTCCCGACTTG |
| *Tnnt2* | TTACATCCAGAAGACAGAGCGG | GTCAATGGCCAGCACCTTCC |
| *Myh7* | GGCAAGACAGTGACCGTGAAG | CGTAGCGATCCTTGAGGTTGTA |
| *Ryr2* | TGCATGAAAGCATCAAACGCA | TCCACCACACAGCCAATCTC |
| *Serca/* *Atp2a2* | CGAACCCTTGCCACTCATCT | CAGGTTCCAGGTAGTTGCGG |
| *Cacna1d* | GGGCAATGGGACCTCATAAATAA | TTACCTGGTTGCGAGTGCATTA |
| *Hey1* | TGGCCAGAAAAAGACGGAGAG | AGCAGATCCCTGCTTCTCAAAA |
| *Nr2f2* | CGCACGAAGGATGTGCTTCTA | TCACACACATAGGGAAAGAGTCA |
| *Myl4* | TCATTGTTTGACCGGACCCC | TTCATCTCTTCAGGCTTGGGC |
| *Mlc2a/Myl7* | GGAGTTCAAAGAAGCCTTCAGC | TCCTCTGGGACACTCACCTT |
| *Sln* | CCTCTTCAGGAGGTGAGGAGAA | AAACAGCTCCCGGGTGTTTA |
| *Hey2* | GCAACAGGGGGTAAAGGCTA | TCAGGTACCGCGCAACTTC |
| *Irx4* | GGCTCCCCAGTTCTTGATGG | CTCGTAGACCGGGCAGTAGA |
| *Myl3* | GCCAAGACAGGAAGAGCTCAAT | CCTCATAGGTGCCTGTGTCC |
| *Mlc2v/Myl2* | TGGGCGAGTGAACGTGAAAA | AGGGTCCGCTCCCTTAAGTT |
| *Shox2* | AACTCCATAAAGGTGTTCTCATAGG | CCTGCTGAAATGGCATCCTT |
| *Hcn2* | CACCTGCTACGCCATGTTCA | CTGGCAGCTTGTGGAAGGA |
| *Hcn4* | GGGAATTCGCAACTGAAGCC | CGGGGTGAGAGGTATCCACA |
| *Nes* | AGGAGAAACAGGGCCTACAGA | GGAGGGTCCTGTACGTGGC |
| *Pax6* | GCGCAGGAGGAAGTGTTTTG | TCTCAGATTCCTATGCTGATTGGT |
| *Afp* | TGTCTGCAGGATGGGGAAAA | GTTCCAGCGTGGTCAGTTTG |
| *Hnf4α* | TGCGACTCTCCAAAACCCTC | TGATGGGGACGTGTCATTGC |

| **Gene name** | **Forward primer** | **Reverse primer** |
| --- | --- | --- |
| *Integrin α1* | CTCACTGTTGTTCTACGCTGC | CGGAGAACCAATAAGCACCCA |
| *Integrin α2* | CACAAAGACACAGGTGGGGT | TGGGATGTCTGGGATGTTGC |
| *Integrin α3* | TGGTCCTAGGTCTGGAGTGG | CAGGGTCAGAAAGAGTGAGGT |
| *Integrin α6* | CCTAACGGAGTCTCACAACTCA | CGGGCATCTGATGTTCACAC |
| *Integrin α7* | TGAAGCAAGGCTGTGGTGAA | CACATCCATGGGCAGAGGTT |
| *Integrin β1* | ACAAATTACCCCAGCCGGTC | CCAGTGGGACACTCTGGATT |
| *Laminin α1* | AGTTTCGAACCTCCTCGCAG | ATGGAACAAGACCTTGCCGT |
| *Laminin α2* | TGAGTATGAAAGCAAGGCCAGA | CTCCAGGGAACATCCTTTGGT |
| *Laminin α3* | AACGTGCCTTCAGGTGACTT | GTTCAGCAAGAGCTGCGAC |
| *Laminin α4* | ACTGCCCAACCATAAGCTGT | CCAGAGGATACGCTCAGCAC |
| *Laminin α5* | GCTGCCAGTAAGGTCAAGGT | AACTTGAGGGCAGTGTAGGC |
| *Laminin β1* | GATTGGACGTCAGTGCAACG | CCACTATGCTAACCCCAGGC |
| *Laminin β2* | TGCTTCCTTTGTGACTCCCG | GTGACCGCAGGGATACCATT |
| *Laminin β3* | ACACACAGCAAGGAAAGGTCC | AGGTCCCCAACAGGTGGATA |
| *Laminin β4* | CCAGCGGCTACGAGAATCAT | TGTGAATGAGCGTGGGACTC |
| *Laminin γ1* | CTAATCCTCGGGGTTGCACA | CGCCACCCATCCTCATCAAT |
| *Laminin γ2* | GCCCAGAAGGTTGATACCAGA | ACTGAGAGGCTGGTCCATCA |
| *Laminin γ3* | GTGCTCCGGGATACAAGAGG | CAGATCCCTGTGTTGGGGTC |
|  |  |  |
